# Supplementary material for: Distinct immune responses associated with vaccination status and protection outcomes after malaria challenge
Source: PLoS Pathog. 2023 May 17;19(5):e1011051. doi: 10.1371/journal.ppat.1011051 (PMC10228810; doi:10.1371/journal.ppat.1011051)
Supplement: S1 Table — (DOCX) [file ppat.1011051.s001.docx]

**S1 Table. Sample availability from the IMRAS trial.**

|  | **Status** | **B0** | | **C0** | | **C1** | | **C6** | | **C7** | | **C112** | |
| --- | --- | --- | --- | --- | --- | --- | --- | --- | --- | --- | --- | --- | --- |
|  |  | **WB** | **PB** | **WB** | **PB** | **WB** | **PB** | **WB** | **PB** | **WB** | **PB** | **WB** | **PB** |
| S002 | P |  |  |  |  |  |  |  |  |  |  |  |  |
| S005 | NP |  |  |  |  |  |  |  |  |  |  |  |  |
| S021 | NP |  |  |  |  |  |  |  |  |  |  |  |  |
| S033 | NP |  |  |  |  |  |  |  |  |  |  |  |  |
| S039 | P |  |  |  |  |  |  |  |  |  |  |  |  |
| S045 | P |  |  |  |  |  |  |  |  |  |  |  |  |
| S049 | Mock |  |  |  |  |  |  |  |  |  |  |  |  |
| S054 | Mock |  |  |  |  |  |  |  |  |  |  |  |  |
| S057 | P |  |  |  |  |  |  |  |  |  |  |  |  |
| S060 | P |  |  |  |  |  |  |  |  |  |  |  |  |
| S061 | NP |  |  |  |  |  |  |  |  |  |  |  |  |
| S062 | Mock |  |  |  |  |  |  |  |  |  |  |  |  |
| S071 | NP |  |  |  |  |  |  |  |  |  |  |  |  |
| S078 | P |  |  |  |  |  |  |  |  |  |  |  |  |
| S104 | Mock |  |  |  |  |  |  |  |  |  |  |  |  |
| S137 | Mock |  |  |  |  |  |  |  |  |  |  |  |  |

WB = Whole blood; PB = Leukapheresis PBMC

Green-highlighted cells indicate available samples
