# Supplementary figures and images for: Distinct immune responses associated with vaccination status and protection outcomes after malaria challenge
Source: PLoS Pathog. 2023 May 17;19(5):e1011051. doi: 10.1371/journal.ppat.1011051 (PMC10228810; doi:10.1371/journal.ppat.1011051)

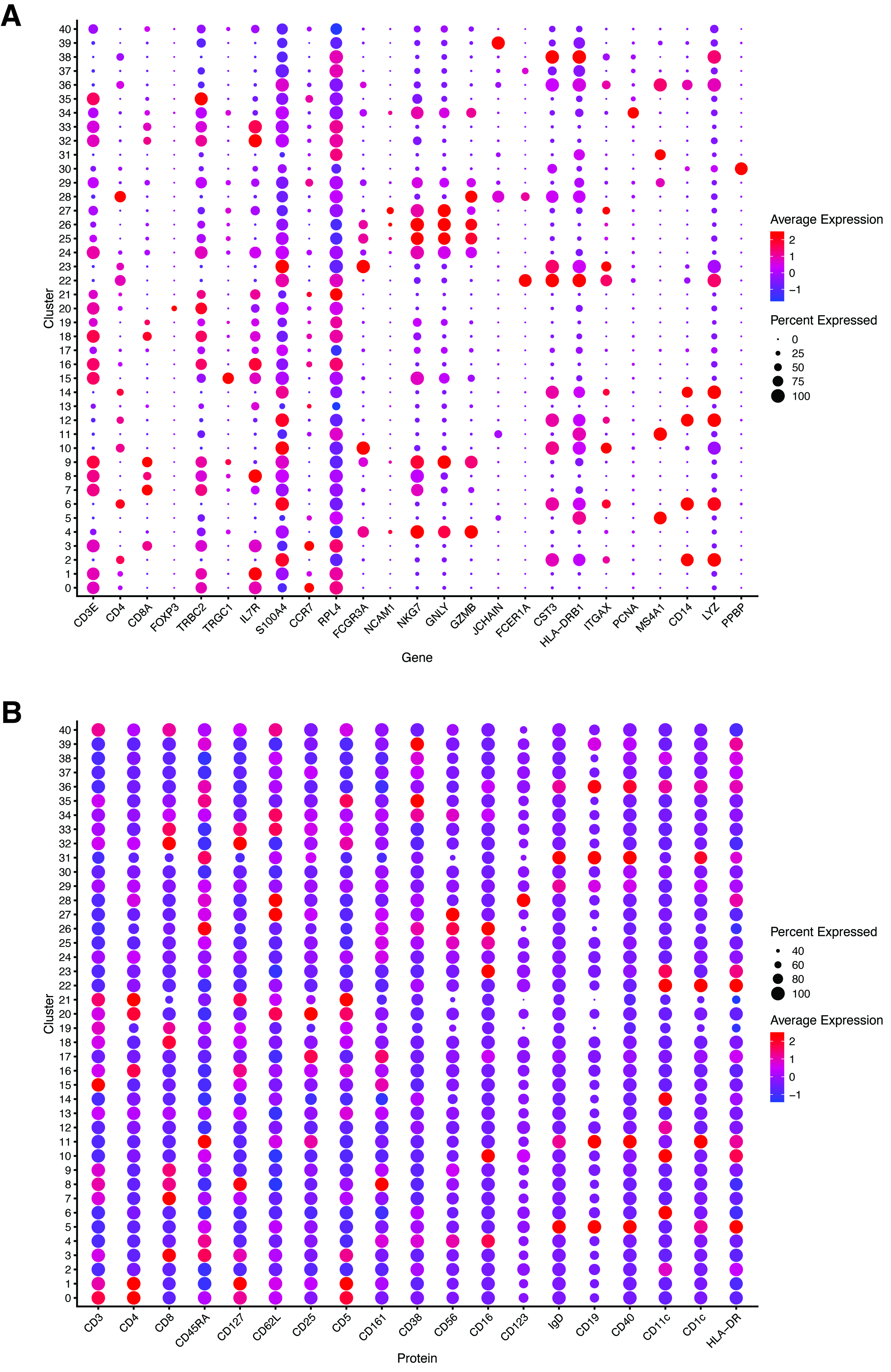

Supplement: S1 Fig — Cell clusters were generated using the Seurat package for weighted nearest neighbor (WNN) clustering on both RNA and ADT data. Each number indicates a distinct cluster with unique A) gene and B) protein expression profile. (TIF) [file ppat.1011051.s008.tif]

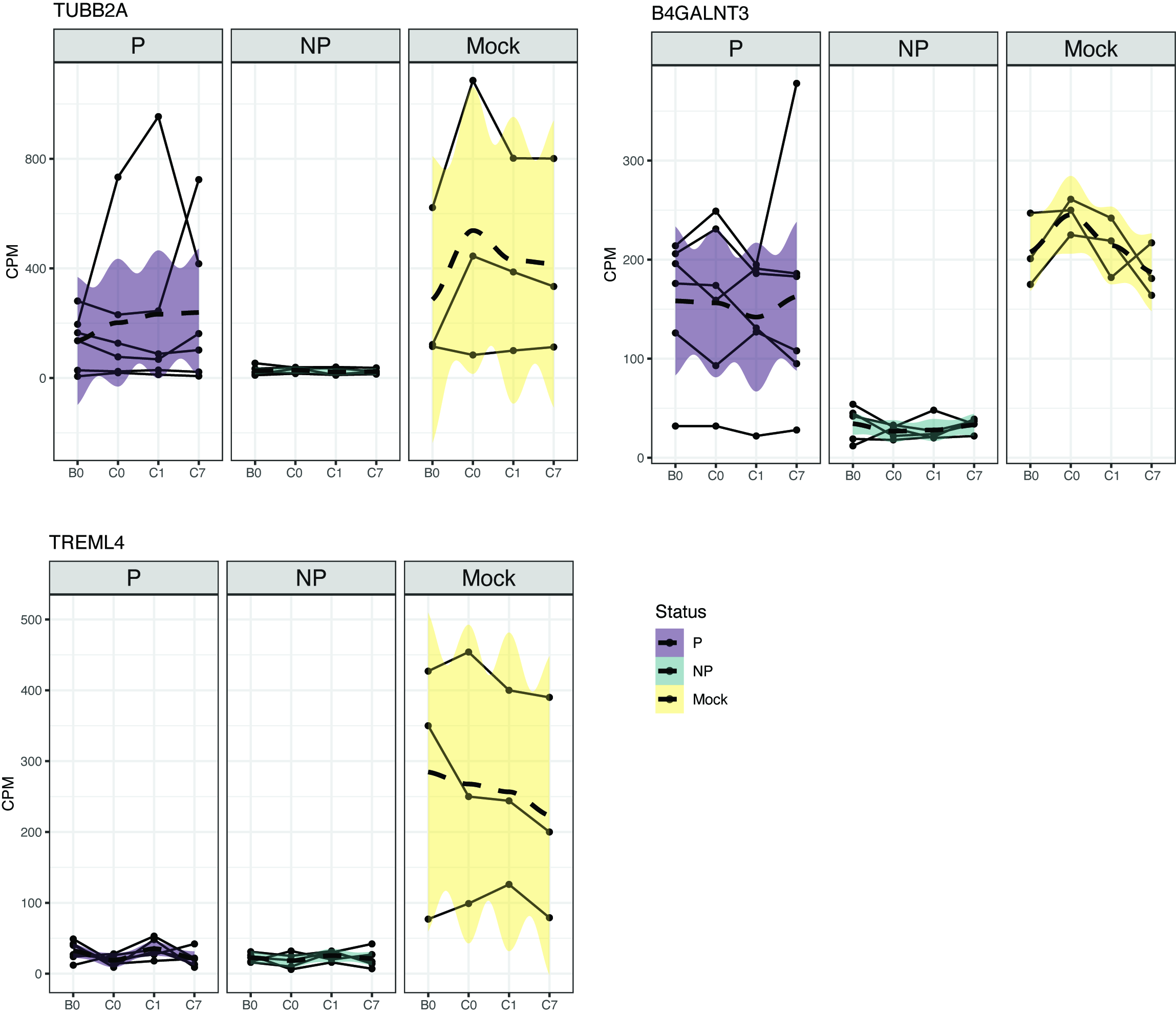

Supplement: S2 Fig — Lines indicate each individual. Dashed lines indicate LOESS regression with 95% confidence interval shown in the highlighted color. Gene expression is in counts per million (CPM) P (n = 6) indicates PfRAS-vaccinated protected individuals, NP (n = 5) indicates PfRAS-vaccinated non-protected individuals, Mock (n = 3) indicates non-infected mosquito bites vaccinated individuals. (TIF) [file ppat.1011051.s009.tif]

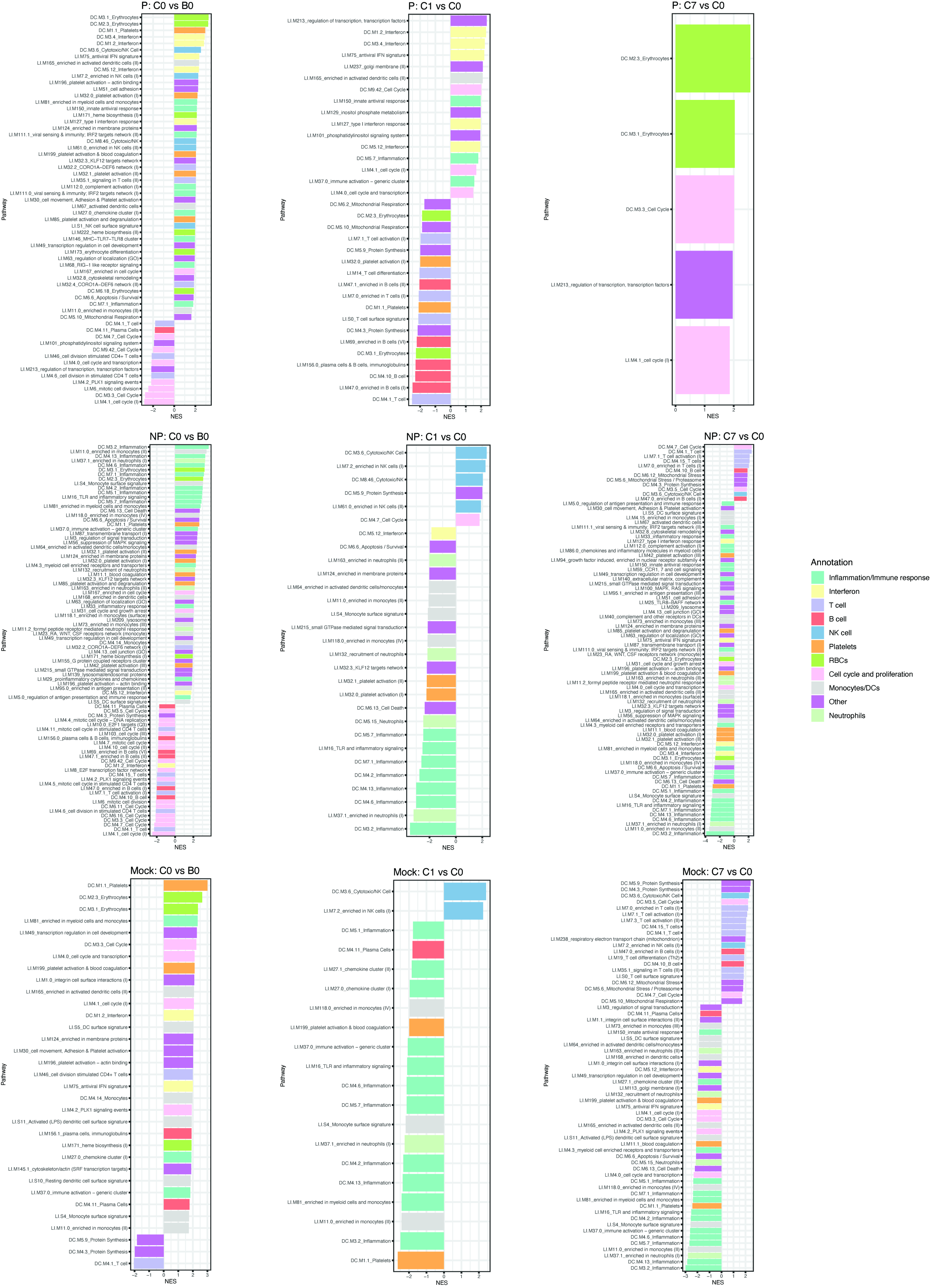

Supplement: S3 Fig — GSEA was analyzed on different comparisons between time points; C0 vs B0, C1 vs C0, C7 vs C0, for each sample group. FDR q-value cutoff was set at 0.01. Each row indicates a gene set module. Bar plots indicate normalized enrichment score (NES) and are colored according to high-level annotations of the gene set modules. (TIF) [file ppat.1011051.s010.tif]

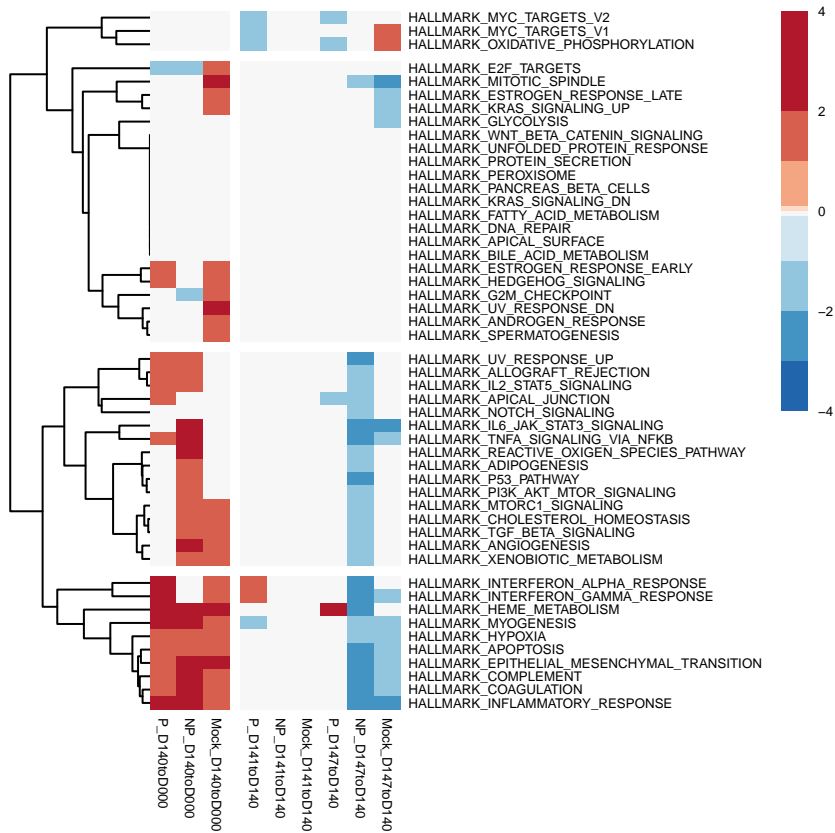

Supplement: S4 Fig — GSEA was performed on comparisons between time points; C0 to B0, C1 to C0, C7 to C0, for each sample group. FDR q-value cutoff was set at 0.01. Each square indicates a gene set module. Color represents normalized enrichment score (NES) obtained from GSEA. Colored row annotations represent high-level annotations of the gene set modules. P (n = 6) indicates PfRAS-vaccinated protected individuals, NP (n = 5) indicates PfRAS-vaccinated non-protected individuals, Mock (n = 3) indicates non-infected mosquito bites vaccinated individuals. (PDF) [file ppat.1011051.s011.pdf]

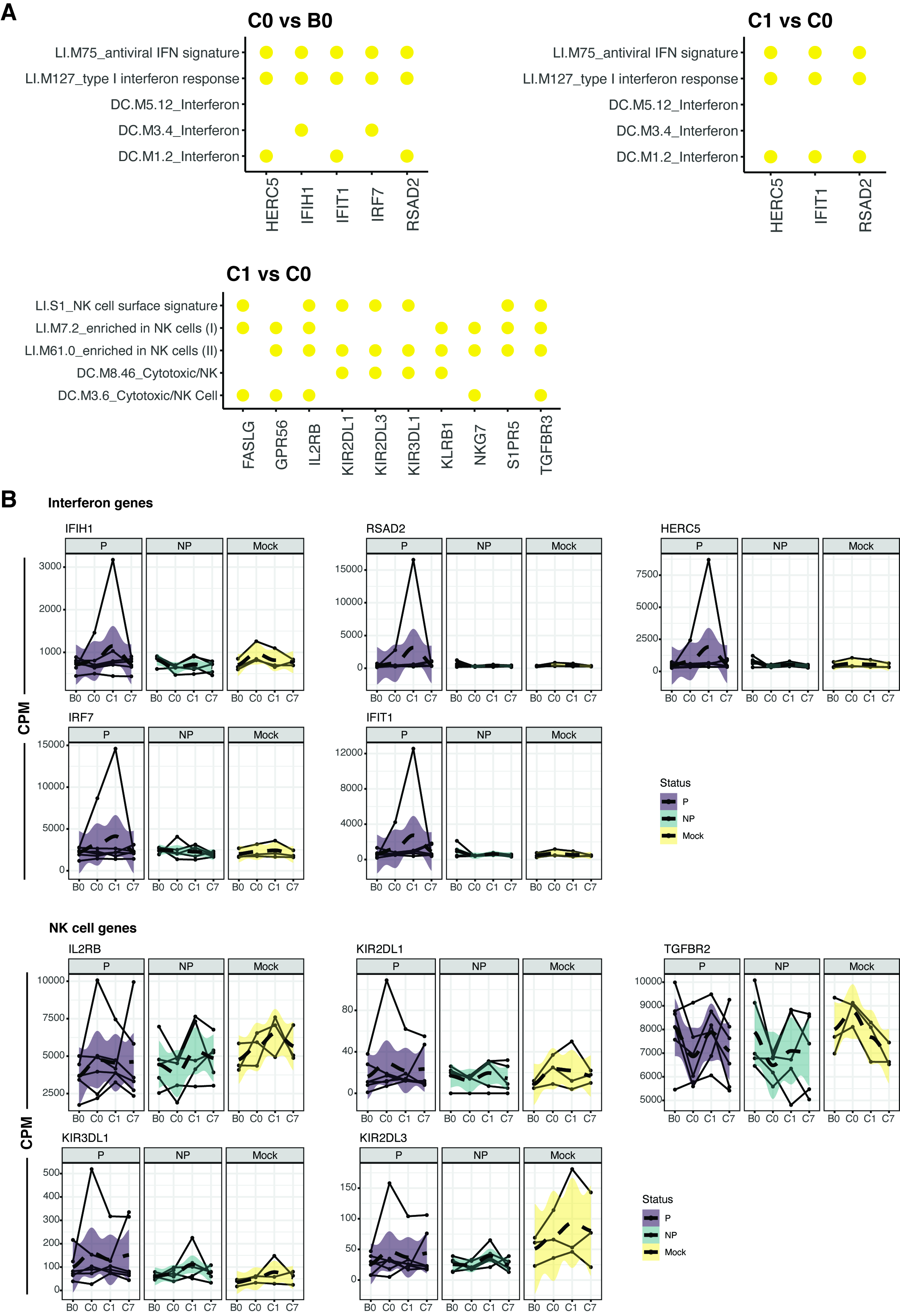

Supplement: S5 Fig — A) Shared leading-edge genes at different time point comparisons for interferon and NK cell-associated BTMs. B) Gene expression levels. Lines indicate each individual. Dashed lines indicate LOESS regression with 95% confidence interval shown in the highlighted color. Gene expression is in counts per million (CPM). P (n = 6) indicates PfRAS-vaccinated protected individuals, NP (n = 5) indicates PfRAS-vaccinated non-protected individuals, Mock (n = 3) indicates non-infected mosquito bites vaccinated individuals. (TIF) [file ppat.1011051.s012.tif]

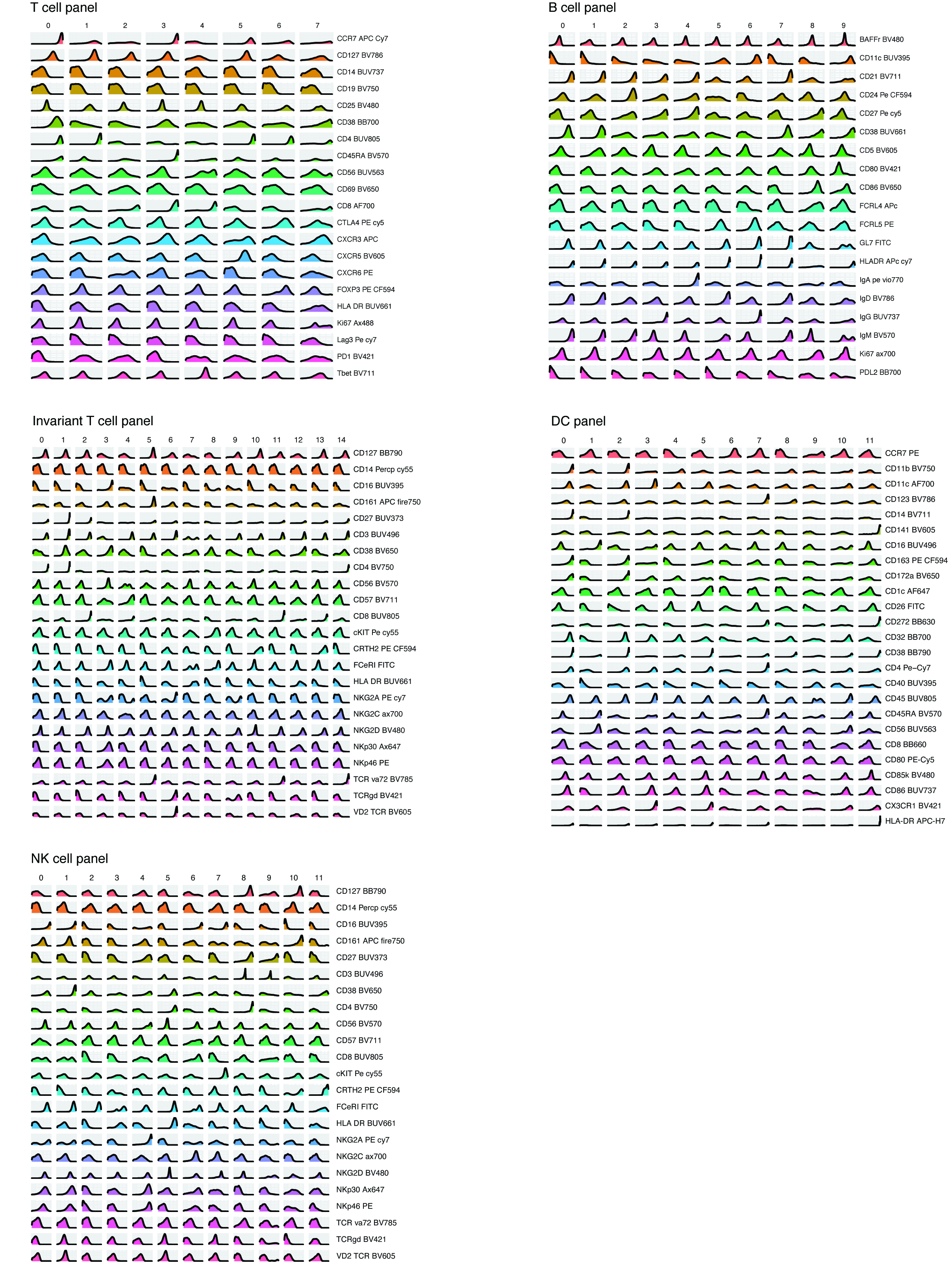

Supplement: S6 Fig — Cell clusters were generated using the Seurat package for unsupervised clustering on each antibody panels. Each number indicates a distinct cluster with unique marker expression profile. Flow cytometry standard (fcs) files were pre-gated to exclude non-relevant cells such as doublets and non-lymphocytes prior to clustering analysis. Additionally, panels were also pre-gated as follows; 1) T cell panel: CD14-CD19-CD3+, 2) B cell panel: CD3-CD19+, 3) invariant T cell panel: CD14-CD19-CD3+, 4) DC panel: CD19-CD3-, 5) NK panel: CD14-CD19-CD3-. The invariant T cell panel and the NK panel originate from the same fcs files and antibody panel. (TIF) [file ppat.1011051.s013.tif]
