# Supplementary material for: Distinct immune responses associated with vaccination status and protection outcomes after malaria challenge
Source: PLoS Pathog. 2023 May 17;19(5):e1011051. doi: 10.1371/journal.ppat.1011051 (PMC10228810; doi:10.1371/journal.ppat.1011051)
Supplement: Attachment — Submitted filename: PPATHOGENS - REBUTTAL_Oyong.docx [file ppat.1011051.s014.docx]

Dear PLOS Pathogens Editors,

Please find enclosed a revised copy of our manuscript “Distinct immune responses associated with vaccination status and protection outcomes after malaria challenge”.

We appreciate the thorough evaluation and feedbacks by the reviewers on our manuscript. We have responded to all reviewers comments and made the appropriate revisions in the manuscript as outlined below. We hope that our manuscript will now be acceptable for publication.

**Part I - Summary**

**Reviewer #1:**

*This manuscript undertakes a transcriptomic analysis of PBMCs from individuals that received irradiated sporozoite vaccination and Plasmodium falciparum challenge. The authors are commended for summarizing the data in easy to digest figures and overall I think this paper provides a useful resource for the malaria vaccine community. I may have missed it but if the authors are making the raw data available for access by other members of the community after publication please include the link to this.*

*It is a little hard through the results as presented but overall this manuscript has been more or less well put together in a logical fashion and I do not have any major issues with what has been presented. There are some points which need a little clarification (below).*

**Response:** We thank the reviewer for their positive reviews of our manuscript. We have previously listed the NCBI GEO number during manuscript submission at Plos Pathogens. To further clarify this, we have added “Data availability” section at Line 720-723.

**Reviewer #2:**

*This is a well-executed study of peripheral blood cell phenotype and gene expression changes during vaccination and challenge infection. The study design allows comparison of vaccinated/protected subjects with vaccinated/unprotected and mock vaccinated subjects; this is (I think) novel and allows the effects of vaccination to be separated from the effects of parasitemia during challenge. As far as I can tell (I am not an expert in some of the techniques employed) the data have been collected and analysed to a high standard. Data interpretation is measured and conclusions have not been overstated. Overall, the findings are in line with expectations from previous studies of system-wide responses to malaria vaccination and CHMI and where there are differences, they have been explored openly and with insight.*

*However, I do fear that we have reached the stage of diminishing returns with this type of study. The inherent limitations of the approach - most significantly that studying peripheral blood tells us very little about the responses we are really interested in (which, in malaria infections, are likely happening mostly in the spleen and liver) and this really limits our ability to move from observation to understanding. The authors are aware of this, and address the issue directly in their discussion, but it really does limit the conclusions that we can draw.*

*For example, the authors state that “Prior to CHMI but after vaccination, immune profiles were relatively similar between the two PfRAS vaccinee groups, protected and non-protected” . So, their data cannot distinguish protective vs non protective responses to vaccination? This suggests (as alluded to above) that the protective responses induced by vaccination are either not cellular/gene expression related or are not manifest in blood. Similarly, post CHMI responses "differ between protected and non-protected/mock" – i.e. they reflect the course of infection rather than the impacts of vaccination? So - which markers are indicative of a protective vaccine-induced response?*

*We now need to take the very many hypotheses that are emerging from such studies and test them experimentally.*

**Response**: We thank the reviewer for their positive comments on our manuscript. We agree with the comment that interpreting results on immunological responses to malaria from peripheral blood can be less informative, given that in the context of PfSPZ vaccination, malaria-specific responses were likely to occur in the liver or lymphoid organs. We have stated this limitation in our discussion in the manuscript. Collecting samples from human tissues poses a major barrier in clinical trials from ethical perspective. In our next project, we aimed to better address this by characterizing immune responses that are specific to *Plasmodium* parasites in the peripheral blood, with the hypothesis that these may reveal, to some extent, similar responses in the tissue organs.

**Reviewer #3:**

*The manuscript by Oyong et al., sought to study the immune mechanisms of protection following vaccination with radiation-attenuated Plasmodium falciparum sporozoites (PfRAS) in a controlled human malaria infection (CHMI) setting. Transcriptional profiling of whole blood and PBMC profiling via high-dimensional flow cytometry analysis was performed on volunteers who received either PfRAS or noninfectious mosquito bites, followed by CHMI. Approximately half of the PfRAS-vaccinated volunteers were protected from blood stage infection following CHMI, allowing for stratification of data based on protection status. The authors identified various gene sets, blood transcriptional modules, and immune pathways that were differentially expression or regulated at different timepoints between the groups (mock vaccinated, protected, and not protected). For example, gene sets associated with interferon responses were increased in protected vaccinees one day following CHMI. The authors performed a considerable amount of data analysis for this manuscript. However, overall, the data are observational, correlative, and not novel (largely confirm data in the literature). More specifically, no functional or mechanistic studies were performed to confirm the observational data. Additionally, while the authors sought to determine correlates of protection, the analyses were so high-level that it was difficult to determine what could possibly be related to protection. (The lack of functional and mechanistic studies also hindered that goal.) Lastly, the analyses largely confirmed existing data in the literature, which strengthens the already published findings, but makes this study less novel. Focusing on certain aspects of the data such as the innate (NK, pDC, monocyte) data or the adaptive (T cell and B cell) data and performing a deeper dive into the existing data along with functional studies would make the study more novel, meaningful, and impactful.*

**Response**: We thank the reviewer for their comments. Detailed responses to reviewer’s comments, including further functional or mechanistic experiments and more elaborate analyses are described below.

**Part II - Major Issues: Key Experiments Required for Acceptance**

**Reviewer #3:**

*• While the manuscript contains a very large set of data, the data are observational and correlative. No mechanistic studies were performed to determine if the observed increase or decrease of certain pathways or gene signatures had functional relevance. For example, to confirm the relevance of increased interferon signaling pathways, IFN-a/b and IFN-g could be quantified in the plasma of study participants. Additionally, functional assays on immune cells could be performed, such as stimulating PBMCs with infected RBCs or PMA/I and quantifying cytokines in the supernatant as well as staining T cells and NK cells for cytokines (eg., IFN-g, TNFa, etc).*

*• Similarly, major findings from the RNA-seq data set should be confirmed by analysis of proteins using other methods (flow cytometry, ELISAs, etc). See bullet point above for analysis of plasma for cytokines and PBMC stimulation assays.*

**Response**: While we agree that additional functional experiments are ideal to confirm findings from our blood transcriptomic data, unfortunately matching plasma time points are not available. In regard to PBMC stimulation experiments, the number of vial aliquots were limited, and we have exhausted all PBMC vials allocated for this study. We have addressed these limitations in our discussion.

*• The study sought to identify correlates of protection, but there appeared to be little emphasis on identifying differences at C0 between the mock, protected and non-protected groups. Data in Fig 3B showed that increases in interferon and NK cell-associated responses were observed in protected vaccinees. A deeper dive into the genes/pathways in those BTMs could provide information on targets for correlates of protection studies or pathways/genes to target for vaccination strategies to boost protection.*

**Response**: We highlighted certain key differences in BTM expressions at C0 between protected, non-protected and mock group in the manuscript. However, our results showed that striking differences in transcriptomic response can be observed one day following CHMI. While we wanted to highlight this diverging response at C1, we understood that selected key responses at C0 (prior CHMI) are important to discuss in relation to malaria protection. We have added extra emphasize on early NK cell response (prior to CHMI) in protected individuals throughout the manuscript. Another sub-paragraph in the results section has been added to identify genes that may play key roles on interferon and NK-cell response. A discussion on some of these genes is also added in the Discussions section.

*• The amount of data included in this manuscript is incredible and the analysis is impressive. However, the descriptions of the data and overall conclusions were high-level. While I can see the importance of keeping all of these data together in one manuscript, it makes it difficult to draw any real meaning. It might be more useful to focus on certain aspects of the data to make functional – and ideally mechanistic – conclusions. For instance, the NK cell data are interesting, along with the pDC, monocyte and interferon data – perhaps a focus on the innate immune response? Or, the T cell and B cell data are interesting, too, especially in relation to the connection with gamma delta T cells and “atypical memory B cells” seen in other malaria studies. Perhaps a deeper dive into the existing data along with some additional functional studies could provide a clearer picture of what’s going on between the mock-vaccinated, protected and not protected groups.*

**Response**: We thank the reviewer for their positive comment on our analysis. As the reviewer pointed out, it is difficult to draw a main conclusion without functional experiments to confirm our findings. However, as responded above, our project is limited from the initial clinical trial design and sample availability to conduct additional confirmatory experiments. Therefore, we aimed to describe the overall systemic immune responses observed from the data and infer possible connections between different types of immune response or cell subsets. For example, we discussed that the decreased of innate immune cells in peripheral blood observed in non-protected and mock groups following CHMI could be due to lack of effective adaptive immune cells that target the parasites and prevent parasite replication. Thus, these innate cells may traffic into the tissues to capture parasites for further antigen presentation. In comparison, the protected group had a decreased of adaptive cells, possibly indicating that these immune cells traffic to the liver to target sporozoites or hepatic merozoites.

**Part III – Minor Issues: Editorial and Data Presentation Modifications**

**Reviewer #1:**

*I think figure 1 would benefit from a visual timeline of the B and C time points – it is explained in the methods and included in Fig S1 but such a figure would be a useful addition to help with interpretation of the figure and I would move figure S1 to the main figure.*

**Response**: S1 Fig is now added to the main figure (Fig 1 – Line 136).

*In the figure in general some of the panels are hard to read as the writing is incredibly small. It would help to try and increase font sizes where possible. (applies particularly to Fig. 1B, Fig. 4A, Fig S4, Fig. S6.*

**Response**: Font sizes have been updated for Fig 1B and Fig 4A. GSEA raw output has been added as a supplementary SData 2.

*In Figure 1 the mock control says 4 of the 5 individuals were selected Why are 4 selected? On what basis?*

**Response**: The 4 mock individuals were selected based on sample availability. Out of the 5 total individuals, 1 individual (S057, S1 Table) had missing samples except at B0, and therefore, we decided to exclude this individual in the analysis.

*In Figure 4 I would change the titles to all be in order (B0 vs C0, C0 vs C1 and C0 vs C7). It is a small point but helps to order the time points for interpretative purposes.*

**Response**: Fig 4 is now updated to barplots for better readability. The order in the updated figure is according to reviewer’s feedback.

Discussion

*Lines 419-420 It may not be a decreased expression of adaptive immunity. What did the adaptive immune response look like? Although the population sizes are different proportionally, is the gene expression also different? Proportion can be driven by expansion of non T/B cells.*

**Response**: Sentence in lines 419-420 refers to transcriptome response – we have edited the sentence for clarify. Regarding cell population size in proportions, it is true that this may reflect cell count changes in other cell subsets. In the case of decreased transcriptome expression of T & B cell associated BTMs in our study, we further confirmed this using ImmunoStates analysis, which provide cell-deconvolution analysis from transcriptomic data. We could not further confirm our transcriptome data with flow cytometry since the sampling time points were different.

*Line 431: Malaria infection does not exist - malaria is a disease caused by Plasmodium infection. Also malaria parasites (Line 495) – it is Plasmodium parasites.*

**Response**: We have reworded the sentence to “malarial infection” and “*Plasmodium* parasites”. This is also edited throughout the manuscript.

**Reviewer #2:**

*Cell frequencies can be misleading - an increase in one subset could be a genuine expansion of that subset or a selective loss of other subsets. Is it possible to calculate (or approximate) absolute cell numbers using CBC data?*

**Response**: While we agree with the reviewer on presenting cell frequency data with proportion is less clear than absolute cell count, we unfortunately did not include cell count beads in the original flow cytometry experiment. We feel that using data from clinical CBC could also be misleading since sample quality; live/dead cells and sample volumes, can considerably varied between samples prior to acquisition in CBC machine vs flow cytometry (considering sample preprocessing, staining, washing).

*The authors claim - in the discussion that their study is “providing important insights into immune mechanisms required to achieve sterilizing immunity” – what exactly are these important insights?*

**Response**: Our study showed that the trajectory of immune response was distinct between protected and non-protected PfRAS vaccinees following CHMI. Immune responses in protected vaccinees were characterized with increased interferon and decreased adaptive immune responses while responses in non-protected vaccinees were associated with decreased inflammation and innate immune responses. While we agree that our results were confirmatory of previous findings, especially in regard to interferon responses, here we showed that such responses diverged as early as one day after CHMI. To ensure that we did not overstate our findings, we have re-worded the sentence in line 430.

**Reviewer #3:**

*• Reference to “interferon responses” is made numerous times, but it is often unclear if it is referring to type I IFN, type II, etc., or if that cannot be determined from the dataset. Either way, that should be clarified.*

**Response**: Manuscript is edited throughout to clarify “interferon responses” observed in our study. Results from GSEA on BTM gene sets showed interferon responses from type I and other non-specified interferons. However, we confirmed that these include type I and type II, specifically IFNa and IFNy, based on GSEA with the Hallmark gene sets.

*• The text in the IPA dataset shown in Fig 4A is nearly impossible to read (had to zoom in to 300% on PDF). The text size should be increased or the data should be shown in a more easily-readable format like a heatmap.*

**Response**: Fig 4A is now presented in a barplot to allow a more readable format. Line 301-319 sentences were edited to adjust with the new figure. Complete information on IPA results is available in S2 Data.

*• While this type of study is important, the relevance to vaccination efforts in malaria-endemic countries is unclear since it’s known that Plasmodium vaccinations have much lower efficacy rates in malaria-endemic countries. Could the authors could make this connection by comparing their RNA-seq data to published RNA-seq data from studies of malaria patients or vaccination studies in endemic countries?*

**Response**: Comparing data from our study to others in malaria-endemic countries is difficult since time points between studies are often unmatched. Here, we highlighted a diverging trajectory of transcriptomic responses as early as 1 day after malaria challenge. To our knowledge, no other PfSPZ vaccine studies in endemic countries have collected and analyzed blood transcriptomic samples at 1 day after challenge, making a direct comparison to our study difficult and less accurate. With samples from natural infection, the time of first primary infection cannot be determined since patients are often enrolled during symptomatic malaria presentation. Nevertheless, our group have compared baseline pre-vaccination responses between various PfSPZ studies, including in malaria naïve and pre-exposed endemic individuals (Neal, NPJ Vaccines, 2022).

*• It is unclear how much the immune profile (transcriptional profile, cellular make-up, etc) in the blood can be used to understand tissue-specific (ie, liver) immune responses. In other words, more data comparing the liver-specific immune response to the circulating immune response – in non-human primate or small animal models – are needed before these types of datasets have much meaning.*

**Response**: We have added this point as a limitation in the discussion (line 687). We agree with the reviewer that interpreting/understanding immune responses in peripheral blood should be taken cautiously and may not reflect immune responses seen in the tissue, of which where parasite infection occur in the pre-erythrocytic stage. We agree that results from animal models can be informative but their interpretation also requires caution due to their immunobiological differences with human. This limitation may be addressed to some degree in the future by analysis of tissue aspirates or timing sample collections to obtain relevant cells during their trafficking.
