# Supplementary material for: Distinct immune responses associated with vaccination status and protection outcomes after malaria challenge
Source: PLoS Pathog. 2023 May 17;19(5):e1011051. doi: 10.1371/journal.ppat.1011051 (PMC10228810; doi:10.1371/journal.ppat.1011051)
Supplement: Attachment — Submitted filename: PPATHOGENS - REBUTTAL_Oyong_V2.docx [file ppat.1011051.s015.docx]

Dear PLOS Pathogens Editors,

Please find enclosed a revised copy of our manuscript “Distinct immune responses associated with vaccination status and protection outcomes after malaria challenge”.

We have responded to Reviewer #2 comments and made the appropriate revisions in the manuscript as outlined below. We hope that our manuscript will now be acceptable for publication.

**Part I - Summary**

*Reviewer #1:*

*The authors have addressed my comments. I think the point was raised that this study is not mechanistic and the limitation of studying PBMCs was raised. Whilst I agree it is not mechanistic and PBMCs give limited information, my personal opinion is that this data represents a valuable and searchable resource for the malaria community. We need to be able to do more mechanistic studies, but we also need to be able to extrapolate form human studies (ie. PBMCs). Yes it confirms some of the existing literature but I think this validates the dataset which can be further mined by the community as new angles of research occur.*

*Reviewer #2:*

*The authors have edited the manuscript to improve the clarity of both the text and the figures, and I thank them for that.*

*The major limitations of the study remain, however, and there are no samples remaining that might allow any experimental exploration of their observations. The decision whether or not to accept the ms for publication is thus down to editorial judgement: are the data presented sufficiently novel and sufficiently useful to others to warrant publication in PLoS Pathogens? I am on the fence about this.*

*One observation however: the conclusion I draw from the authors responses to some of the reviewer comments is that the very early (within 24h) innate immune response influences the outcome of CHMI and this early response is heterogeneous between subjects both after immunisation but also, crucially, before immunisation (or in the absence of immunisation). In other words, underlying heterogeneity in innate immune responses (possibly linked to NK/KIR, but this is not proven) affects the outcome of CHMI and may also affect the outcome of immunisation. I am reminded of a paper published almost 20 years ago (Walther et al, Immunity 2005 Sep;23(3):287-96. doi: 10.1016/j.immuni.2005.08.006.) that came to similar conclusions - that the immediate innate response drives either control of parasitemia or control of the fever response after CHMI - and I wonder whether this conclusion needs to be stated even more clearly in the abstract and discussion of this present manuscript? The relevance of this observation is not only that it may explain the heterogeneous response to vaccination in the US volunteers but may also underlie population level differences in vaccine efficacy?*

**RESPONSE:**

We agree that the heterogeneity of innate response could have detrimental impacts in the outcome of sterilizing malaria immunity. In our study, we observed an increased in NK-cell related gene set modules in protected vaccinees prior to malaria challenge (CHMI), but after PfRAS vaccination. As discussed in the manuscript, it is unknown if this innate NK cell response is due to PfRAS vaccination, indicating adaptive NK-cell response, or inherent genetic differences in the volunteers. It is important to note that we did not specifically compare differences prior to vaccination, due to limited sample size in our cohort. Other data from our group suggested that baseline immune differences could impact protection outcomes (Neal *et al*., npj Vac, 2022), albeit there was no evidence that NK cell response was the main mediator. We therefore refrain to conclude or overstate that the heterogeneity of NK cell response is due to inherent differences, since indirect cytokine signaling from the adaptive compartments (which is due to vaccination) could also influence NK cell response prior to CHMI in protected vaccinees.

In agreement to the reviewer, we edited the abstract and discussion to highlight the importance of early NK cell response to malaria protection, and that this could be explained by genetic differences in population. We also added the useful reference that the reviewer suggested in the discussion.

*Reviewer #3:*

*The revised manuscript by Oyong et al., describes immune mechanisms of protection following vaccination with radiation-attenuated Plasmodium falciparum sporozoites (PfRAS) in a controlled human malaria infection (CHMI) setting. Transcriptional profiling of whole blood and PBMC profiling via high-dimensional flow cytometry analysis was performed on volunteers who received either PfRAS or noninfectious mosquito bites, followed by CHMI. Approximately half of the PfRAS-vaccinated volunteers were protected from blood stage infection following CHMI, allowing for stratification of data based on protection status. The authors addressed this reviewer’s concerns adequately and provided additional analyses that strengthen their conclusions. Overall, this manuscript includes important data that will shed light on immune mechanisms of protection following whole sporozoite Plasmodium vaccination.*

**Part II – Major Issues: Key Experiments Required for Acceptance**

*Reviewer #1: (No Response)*

*Reviewer #2: Amend abstract and discussion to really stress the pivotal role of early innate responses (and the heterogeneity therein) in the outcome of CHMI and vaccination.*

**RESPONSE:**

We have made some changes in abstract and discussion to address reviewer’s suggestion on the importance of early responses on the outcome of malaria immunity.

*Reviewer #3: none*

**Part III – Minor Issues: Editorial and Data Presentation Modifications**

*Reviewer #1: (No Response)*

*Reviewer #2: (No Response)*

*Reviewer #3: none*
